# Impact of snus use in teenage boys on tobacco use in young adulthood; a cohort from the HUNT Study Norway

**DOI:** 10.1186/s12889-019-7584-5

**Published:** 2019-09-13

**Authors:** Liv Grøtvedt, Lisa Forsén, Inger Ariansen, Sidsel Graff-Iversen, Turid Lingaas Holmen

**Affiliations:** 1 Department of Health and Inequality, Norwegian Institute of Public Health, PO Box 222, Skøyen, N-0213 Oslo, Norway; 2 Department of Chronic Diseases and Ageing, Norwegian Institute of Public Health, Oslo, Norway; 3 Norwegian National Advisory Unit on Women’s Health, Oslo University Hospital, Oslo, Norway; 4 Department of Public Health and General Practice, HUNT Research Center, Norwegian University of Science and Technology, Levanger, Norway

**Keywords:** Snus, Smokeless tobacco, Smoking, Dual use, Tobacco, Tobacco harm reduction

## Abstract

**Background:**

As smoking rates decreased, the use of Swedish snus (smokeless tobacco) concordantly increased in Norway. The role of snus as possible contributor to the reduction of smoking has been widely discussed. Our aim was to quantitate transitions in snus use, smoking and dual use of snus and cigarettes in a young male population.

**Methods:**

This prospective cohort study includes 1346 boys participating in the Nord-Trøndelag Health Study in Young-HUNT1 1995–97, age 13–19 and in HUNT3 2006–08, age 23–30. Participants reported on tobacco use at both points of time. Models with binominal regression were applied to examine relative risks (RRs), of adolescent ever snus users, dual users or smokers (reference: never tobacco use), to be current snus only users, smokers (including dual users), or tobacco free in adulthood.

**Results:**

Current tobacco use in this male cohort increased from 27% in adolescence to 49% in adulthood, increasing more for snus only use and dual use than for smoking only.

The adjusted RR (95% CI) of becoming a smoker as young adult, was 2.2 (CI 1.7–2.7) for adolescent snus users, 3.6 (CI 3.0–4.3) for adolescent dual users, and 2.7 (CI 2.2–3.3) for adolescent smokers. RR to become snus only users as adults was 3.1 (2.5–3.9) for adolescent dual users, 2.8 (2.2–3.4) for adolescent snus users and 1.5 (1.0–2.2) for adolescent smokers. The adjusted RR for the transition from adolescent tobacco use to no tobacco use in adulthood was similar for snus users and smokers with RR 0.5 (CI 0.4–0.7), but considerably lower for dual users with RR 0.2 (CI 0.2–0.3).

**Conclusions:**

The use of snus, with or without concurrent smoking, carried a high risk of adult smoking as well as adult snus only use. Dual use seemed to promote the opportunity to become snus only users in adulthood, but made it also more difficult to quit. The benefit of snus use for harm reduction is not evident in our cohort, as the combination of smoking and dual use resulted in high smoking rates among the young adults.

## Background

The smokeless tobacco (ST) sold in Norway is an unfermented, moist tobacco product, known as snus. Snus use started to increase after 1990, first among young men, and from 2005 among young women [1]. In 2017, 25% of young men and 14% of young women used snus daily (age 16–24 years). Smoking rates in Norway have declined steeply since the millennium. Among young people, daily snus use (19%) is now more common than daily smoking (3%) [2, 3]. Young men and women using both products, 13 and 7% respectively, most often use snus daily and cigarettes occasionally [3].

Smoking is responsible for one fifths of all premature deaths before the age of 70 in Norway [4]. The harmful effects of smoking are well known, and it is a broad agreement that snus use is less harmful than smoking. The snus used in Scandinavia is known to have relative low content of tobacco-specific nitrosamines, corresponding to a lower cancer risk, compared to some of the products marketed in USA [5, 6]. Snus is highly addictive due to its nicotine content, and health effects, such as higher mortality among patients with cardiovascular disease, increased risk of type 2 diabetes and increased risk of premature birth and stillbirth, are found among snus users [7–10].

Since the ban on advertising tobacco products in Norway in 1975, a variety of tobacco control policies were implemented, with the prohibition of smoking in restaurants and bars regarded as one of the most effective (2004). In areas where smoking is restricted, snus use may ease withdrawal symptoms and maintain the dependence on cigarettes [6], or enhance the change to snus only use. The increased use of snus in Norway already from the late nineties may have been influenced by a shift from loose moist snus to the more convenient and modern portion snus with added flavors. In Norway and Sweden, snus use among adults has been associated with smoking cessation rather than smoking initiation, as more men switched from cigarettes to snus than from snus to cigarettes. Among the snus users, however, a majority continued their snus use rather than quitting tobacco altogether [11–13]. Decreasing smoking and increasing snus use is described among Norwegian adolescents 2002–2010 [14].

The present study investigated transitions in tobacco use in a comprehensive approach within a cohort population. Our main objective was to assess the associations between snus use, including dual use, in adolescence and tobacco use in young adulthood 11 years later, using a cohort study where adolescent tobacco use could be followed up into adulthood.

## Methods

### Baseline and follow-up surveys

**Fig. 1:**
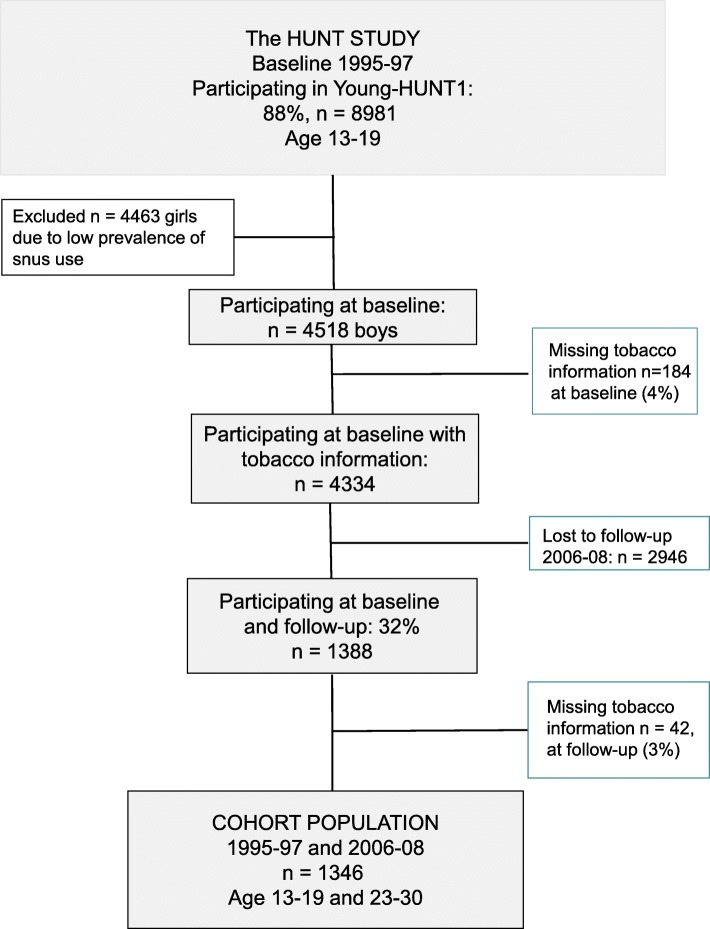
Flow Chart. Participants with longitudinal questionnaire data from Young-HUNT1 and HUNT3

The Nord-Trøndelag Health Study (HUNT) is a large population based health study conducted regularly in the county of Nord-Trøndelag since 1986. All inhabitants 13 years and older are invited to participate. All students in junior high school (age 13–16 years) and high school (16–19 years) were invited to the Young-HUNT1 survey during 1995–97. Young-HUNT1 was the baseline for our cohort study. A total of 8981 adolescents (88% of all invited) participated. Self-reported questionnaires in the Young-HUNT1 survey were completed in schools in an exam setting. The Young-HUNT1 participants were later included in the HUNT3 survey (2006–08) as young adults aged 23–30 years, giving an 11 years follow-up. The main questionnaire in HUNT3 was delivered by post, and collected in person, when participants attended the health examination part of the survey. As the young adults in our study were part of a large study among all adults in the county, many may have moved out of the county for further education and thus were not eligible for invitation to the HUNT3 survey. A low participation rate among young adults was partially offset by a short non-responder survey by mail [15, 16]. All participants gave written consent, in addition to consent from the parents/ guardians for those under the age of 16 years in Young-HUNT1. As shown in the flow chart in Fig. 1, only boys were selected for analysis in the present study because of low baseline prevalence of snus use among girls (3%). However, comparative key results for girls are shown in Additional file 1.

Of the 4334 boys participating in the Young-HUNT1 survey with tobacco information, 1388 (32%) also participated in the HUNT3 survey as young men. Of those, 1346 participants responded to the questions about tobacco at both time points and constituted the present study population (Fig. 1). Missing values for both tobacco questions constituted 4% in adolescence and 3% in adulthood, not included in the study population of 1346 participants.

### Tobacco use measures

The questions about tobacco use were similar at both points of time and were used to construct both the predictors and the outcome variables in the analyses. The main question about smoking was worded “*Do you smoke, or have you ever smoked?”* The response categories were: *No, never; Yes, but I have quit; Yes, occasionally; Yes, every day.* The question about snus was worded *“Do you use, or have you ever used snus, chewing tobacco or similar products?”* with the response categories as for smoking. We defined the baseline tobacco status into four mutually exclusive groups of *ever smokers* (current or former, no snus), *ever snus users* (current or former, no smoking), *ever dual users* (both snus and smoking, current or former), and *never-users* of any of these products. Former tobacco use was included in the predictor variables as ever use, instead of using separate covariates to address former use. Both daily and occasional use were included in ever and current tobacco use in the main analyses. Quantity of tobacco use was only given for the daily users, and is therefore not included in the analyses. Other tobacco products apart from cigarettes and snus were rarely used in Norway and not asked for in the survey [17].

### Sociodemographic and behavioural measures

The questions were worded as below and the categories are given in Table 1. Parents Co-residence: “*Are your parents separated or divorced?”* Family smoking: “*Does anybody in your home smoke*?” Adolescents’ self-reporting of parental alcohol use: “*Have you ever seen any of your parents drunk?*” The pupils’ plans for own education were asked: *“What are your further educational plans?”* All included covariates were measured at baseline, and had mutually exclusive categories. Variables with a theoretical causal association to both the predictor and the outcome (confounders), and with a notable impact on the effect measure, were included in the main multivariable analyses. The factors considered were parents’ status of co-residence, parental divorce, family smoking and parental alcohol use. Family smoking may also act as a proxy for parental socioeconomic status [18–22]. Parents’ alcohol use may influence parental monitoring and in turn adolescent smoking [19].

**Table 1:** Sociodemographic and behavioural characteristics, by current tobacco use in adolescence^a^

	Total	Snus use, but not smoke	Smoking, but not snus	Dual use	No tobacco	*p*-value^**^
Participants	1346 (100)	149 (11)	105 (8)	109 (8)	983 (73)	
Age years, mean ± SD	16.2 ± 1.8	17.0 ± 1.6	16.8 ± 1.6	16,7 ± 1.5	15.9 ± 1.8	< 0.001
13–15 years, n (%)	625 (100)	40 (6)	32 (5)	33 (5)	520 (83)	
16–19 years, n (%)	721 (100)	109 (15)	73 (10)	76 (11)	463 (64)	< 0.001
Parents living together						
Mother and father married/ living together, n (%)	1116 (100)	118 (11)	80 (7)	81 (7)	837 (75)	
Mother and father divorced/ not living together, n (%)	187 (100)	29 (16)	23 (12)	25 (13)	110 (59)	< 0.001
Family smoking						
No family member smoke, n (%)	685 (100)	71 (10)	35 (5)	31 (5)	548 (80)	
Father *or* mother smokes, n (%)	362 (100)	41 (11)	39 (11)	39 (11)	243 (67)	
Father *and* mother smoke, n (%)	235 (100)	30 (13)	22 (9)	29 (12)	154 (66)	
Siblings *and/ or* others smoke, but no parent, n (%)	59 (100)	7 (12)	8 (14)	10 (17)	34 (58)	< 0.001
Parental alcohol use						
Have never seen parents drunk, n (%)	507 (100)	21 (4)	21 (4)	21 (4)	444 (88)	
Yes, a few times, n (%)	451 (100)	59 (13)	42 (9)	42 (9)	308 (68)	
Yes, sometimes a year, monthly or weekly, n (%)	347 (100)	65 (19)	39 (11)	45 (13)	198 (57)	< 0.001
Plans for own education						
Not yet decided, n (%)	403 (100)	36 (9)	37 (9)	30 (7)	300 (74)	
Vocational high school or similar, n (%)	420 (100)	54 (13)	33 (8)	44 (10)	289 (69)	
High school until 4 years, n (%)	237 (100)	25 (11)	16 (7)	15 (6)	181 (76)	
University, more than 4 years, n (%)	242 (100)	30 (12)	17 (7)	17 (7)	178 (74)	< 0.301

^a^ All the tobacco use categories include both daily and occasional use. Variables with missing data include Parents living together (3%), Family smoking (0.4%), Parental alcohol use (3%), and Plans for own education (3%). ^**^ *p*-value: test for independence between the socio-demographic and the tobacco variable at baseline

Information on personality traits and school functioning were considered as potential confounders (sensitivity analyses). An 18-item version of the Eysenck Personality Questionnaire, scored according to the established guide, was used to measure three dimensions of personality introversion-extraversion, neuroticism and psychoticism, and included as covariates in the multivariable regression model [23]. Tobacco use is known to be associated with all three dimensions of personality traits [24, 25]. Psychosocial and behavioral factors were considered as confounding regarding tobacco transistions from adolecence to adulthoood. The adolescents were also asked to consider 13 statements concerning school functioning, evaluated on a 4-point scale ranging from «never» to «very often». Main themes for the 13 items are academic and conduct problems, and lack of joy in school, with the three dimensions “gratuitous”, “restless, quarrelsome” and “well-adjusted, positive [26].

### Statistics

The three outcome variables of *current* tobacco use in young adulthood (follow-up), were: 1) current smoking or dual use versus “no” tobacco use (*N* = 1050, due to the omitted adult snus users), 2) current snus only use versus “no” tobacco use (*N* = 988, due to the omitted adult smokers and dual users) and 3) no current tobacco use versus “any” tobacco use (*N* = 1346, no cohort participants omitted). The predictor tobacco variables in adolescence (baseline) were *ever* snus use, *ever* smoking and *ever* dual use, all versus never tobacco use, as mutually exclusive groups.

Pearson’s Chi-square or ANOVA were used in bivariable analyses. In multivariable regressions, we used a log-risk model with binreg (binomial family) in STATA and chose log as link-function, giving the outcome RR. Convergence problems occurred when more covariates than age were included. Binreg was then replaced with a log-risk model, poisson family (GLM), with the option robust. This was treated as binomial regressions with RRs, with somewhat increased standard errors (SE). STATA (version 15) was used.

Among the possible confounders mentioned in subsection above, only family smoking (dichotomized into no vs any family smoking) altered the RRs, and were thus included in the main analyses. Personality traits and school functioning were included as confounders in sensitivity analyses. Factor analyses were performed to achieve the dimensions for the Eysenck Personality and school functioning scales.

## Results

### Study participants

Mean age for the 1346 study participants as adolescent boys was 16.2 (range 12.7–20.2) years and 27.9 (range 23.0–33.1) years as young adults. While 27% of the boys were *current* tobacco users in adolescence (Table 1), 33% were *ever* tobacco users (Table 2). Among the young adults 49% were current tobacco users.

**Table 2:** Tobacco use in two age groups in adolescence and adulthood. Number (%). Study population, unadjusted

Current tobacco use as young adults					
	No tobacco	Snus only	Smoke only	Dual use	All
Ever tobacco use age 13–15					
No tobacco	260 (54.4)	87 (18.2)	57 (11.9)	74 (15.5)	478 (100)
Snus only	6 (12.5)	21 (43.8)	8 (16.7)	13 (27.1)	48 (100)
Smoke only	14 (31.1)	7 (15.6)	14 (31.1)	10 (22.2)	45 (100)
Dual use	7 (13.0)	18 (33.3)	6 (11.1)	23 (42.6)	54 (100)
All	287 (45.9)	133 (21.3)	85 (13.6)	120 (19.2)	625 (100)
Ever tobacco use age 16–19					
No tobacco	313 (73.1)	74 (17.3)	23 (5.4)	18 (4.2)	428 (100)
Snus only	24 (35.3)	46 (40.0)	8 (7.0)	12 (10.4)	115 (100)
Smoke only	49 (42.6)	11 (16.2)	20 (29.4)	13 (19.1)	68 (100)
Dual use	19 (17.3)	32 (29.1)	23 (20.9)	36 (32.7)	110 (100)
All	405 (56.2)	163 (22.6)	74 (10.3)	79 (11.0)	721 (100)

Among the boys participating at baseline, one in three participated in our study population. Excluded participants that only attended the baseline examination but not follow-up had higher prevalence of adolescent tobacco use (33% vs 27%), family smoking (57% vs 49%), and parental divorce (22% vs 14%) than the study population (Additional file 2). We found no corresponding difference in adolescents’ experience of alcohol use among the parents. Also, no difference was found in the pupils educational plans between participants and non-participants to the study population. The difference in attendance between younger and older age adolescent tobacco users is shown in Additional file 3. Among older adolescents (16–19 years) the prevalence of smoking was 16% in the group that did not attend follow-up, compared to 10% in the study population. Smaller and non-significant differences were found among the younger adolescent (13–15 years) smokers, snus users and dual users.

Within our cohort 51 participants gave inconsistent answers about their smoking behavior at baseline and follow-up. They reported “never smoked” as adults, while 8 of them reported daily smoking and 43 occasional smoking as adolescents. Regarding snus use, 53 participants stated at follow-up that they had never used snus, but reported occasional snus use at baseline. In addition, 25 participants reported “never snus use” at follow-up, but reported to have quit snus at baseline. In additional analyses where participants with inconsistent answers were removed, the main results were confirmed, but with larger effect size of the transitions of tobacco use. The effect size of the transitions from tobacco use to no tobacco use decreased when removing inconsistent answers (data not shown).

### Bivariable analyses

The prevalence of tobacco use in the study population doubled from adolescents aged 13–15 to those aged 16–19. All adolescent tobacco use categories had higher prevalence of parental divorce, family smoking and parental alcohol use than adolescent no tobacco users. Level of educational plans did not differ significantly between adolescent tobacco users and no tobacco users (Table 1). Adolescent smokers and dual users, but not snus users, had higher mean levels of neurotic personality traits than non-tobacco users. All categories of adolescent tobacco users had higher mean levels of extrovert personality traits, while only dual users had higher levels of psychotic personality traits than the non-users of tobacco. Among school factors, only the factor measuring the dimension “restless, quarrelsome” showed higher mean levels of problems across all tobacco use categories, compared to non-use of tobacco (Additional file 4).

**Fig. 2:**
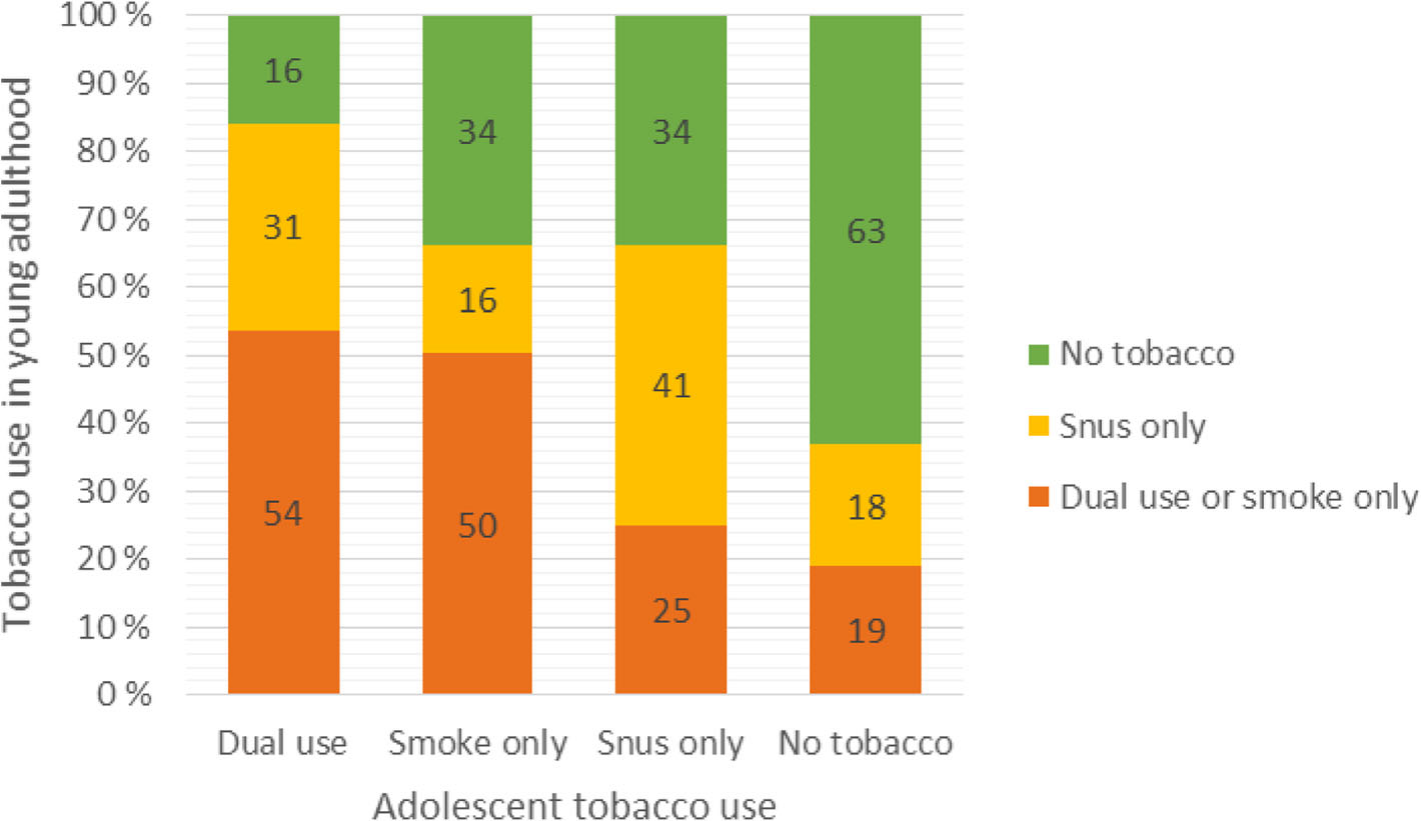
Tobacco use as young adults within adolescent tobacco user groups. Percent. Study population, unadjusted

The crude prevalence of ever tobacco use in adolescence and current tobacco use in young adulthood are shown in Fig. 2 and Table 2. Most of the adolescent snus users were still snus users as adults (41%), 34% had quit all tobacco and 25% had become smokers (including dual users). Among the adolescent smokers, 50% were still smokers or dual users in adulthood, 34% had quit all tobacco use and 16% had become snus users. Among the adolescent dual users, 54% had become smokers or dual users as adults, 31% had become snus only users and 16% had quit all tobacco use. Hence, the probability for young ever tobacco users to quit was about one in three for snus users and smokers, and about one in six for dual users. Among the dual users, however, nearly one third had quit smoking and switched to snus only.

The youngest tobacco users at baseline (13–15 years) had a high prevalence of current tobacco use in young adulthood (Table 2). Among the youngest snus users (13–15 years), 44% had become smokers or dual users as adults, compared to 17% among the older adolescent snus users (16–19 years). In both age groups about 40% maintained their snus-only use into adulthood. For the transition from adolescent smoking to adult snus only use, as well as from dual use to snus only use, small differences were found between age groups.

The dual users were mostly a mix of daily users of one tobacco product and occasional users of the other product, besides a few using either both products occasionally or both products daily. This was examined in subgroups of current duals users within the study population in adolescence (*N* = 109) and adulthood (*N* = 199). The composition of the dual user group changed from adolescence to adulthood. In adolescence, the majority of dual users (42%) were daily smokers and occasionally snus users, versus 25% in adulthood. In adulthood, the majority of dual users (52%) were daily snus users and occasionally smokers, versus 21% in adolescence (data not shown).

In our study population, one third of the initial *occasional* smokers and snus users had quit all tobacco, while half of them were daily users as adults, regarding current use of tobacco at both time points. For *daily* smokers and snus users, 4 and 17% were quitters, and 90 and 70% respectively, were daily users of one of the products in young adulthood. Altogether, current daily tobacco use increased from 12% in adolescence to 36% in young adulthood. Occasional tobacco use decreased from 15 to 13% (data not shown).

### Multivariable analyses

Table 3 shows the results from the multivariable regression analyses of associations between ever tobacco use in adolescence, and risk of current or no tobacco use in adulthood. The RRs with confidence intervals (CI) of adolescent snus users to be smokers in young adulthood, adjusted for age and family smoking, was 2.2 (1.7–2.7) (Table 3). The RRs of adolescent smokers and dual users of still being smokers in adulthood, adjusted for age and family smoking, were 2.7 (2.2–3.3) and 3.6 (3.0–4.3), respectively. Adolescent snus users and smokers had a doubled, or more than doubled, likelihood to be smokers as adults. Adolescent dual users carried more than a threefold risk to be smokers as young adults, but at the same time also a comparable risk to be snus only users.

**Table 3:** Tobacco use in adulthood according to adolescent ever tobacco use 11 years earlier. RR (CI)

	Current smoking/dual us as young adults^a^		Current snus only use as young adults^a^		No tobacco use as young adults^b^	
	Adjusted for age	Adjusted for age and family smoking	Adjusted for age	Adjusted for age and family smoking	Adjusted for age	Adjusted for age and family smoking
	*N* = 1050	*N* = 1046	*N* = 988	*N* = 984	*N* = 1346	*N* = 1341
Tobacco use at baseline:						
No tobacco	ref.	ref.	ref.	ref.	ref.	ref.
Snus use	2.18 (1.67–2.85)	2.15 (1.69–2.73)	2.87 (2.32–3.55)	2.78 (2.24–3.44)	0.50 (0.40–0.62)	0.49 (0.40–0.61)
Smoking	2.59 (2.12–3.16)	2.68 (2.15–3.34)	1.51 (1.02–2.26)	1.47 (0.98–2.22)	0.50 (0.38–0.64)	0.52 (0.40–0.68)
Dual use	3.02 (2.56–3.55)	3.61 (3,04–4.30)	3.04 (2.51–3.69)	3.14 (2.53–3.89)	0.23 (0.16–0.33)	0.24 (0.16–0.34)

^a^ Versus no current tobacco use. ^b^ Versus any tobacco use

Adolescent snus users had nearly a threefold risk of still being snus users as young adults, with adjusted RR 2.8 (2.4–3.4). Adolescent smokers had no significant likelihood of being snus only users as young adults. The likelihood of adolescent boys to become tobacco free in young adulthood, given tobacco use in adolescence, was comparable for previous snus users and smokers with RR 0.5 (0.4–0.7). The adolescent dual users had clearly the lowest likelihood to become tobacco free in young adulthood (Table 3).

### Sensitivity analyses

There were theoretical reasons for including “school factors” and “personality traits”, as confounders in the analyses. Due to the relatively high rates of missing values attached to these variables (Additional file 4), we chose to present the results as sensitivity analyses. These sensitivity analyses gave weaker associations, but no substantial change from the main results in Table 3: With all confounders included, the risk for adolescent snus users to be current smokers as adults were RR 1.9 (1.4–2.6). The corresponding RRs for smokers and dual users to be current smokers at follow-up were 2.5 (1.9–3.3) and 3.1 (2.5–3.9). The fully adjusted risks of adolescent snus users, smokers and dual users to be current snus users as adults were RR 2.5 (1.9–3.1), RR 1.3 (0.9–2.1) and RR 2.7 (2.2–3.5).

### Key results for females

A few comparative results for women belonging to the same cohort are given in Additional file 1. Current tobacco use was 22% in adolescence and 31% in adulthood, with cigarettes as the main product at baseline (20%) and follow-up (22%). Current snus only use was 2% in adolescence and increased to 6% in adulthood; while 3% were dual users at both time points.

## Discussion

In this study, adolescent snus only users conferred a doubled risk of smoking, and almost a threefold risk to continue with snus as young adults. Adolescent dual users conferred a threefold risk to still be smokers in adulthood. The transition from smoking to snus only use was less common. Any adolescent tobacco use was associated with increased risk of smoking, including dual use, 11 years later.

### Transitions between tobacco products

The associations between adolescent snus use and smoking in young adulthood in this study were similar to previous studies [27–29]. One recent study among young men enrolled in the army in Switzerland did not find any beneficial effect of snus use on smoking, but increased likelihood of smoking initiation and continuation [30]. A Swedish study found, similar to the present study, adolescents’ progression in tobacco use mainly to be associated with mixed use of cigarettes and snus [31]. In our study, a considerable proportion of the adult dual users used snus daily and smoked occasionally, instead of the opposite constellation, in line with another Norwegian study [14]. A US review including six studies among both adolescents and adults published since 2000 demonstrated the heterogeneity in design across studies, but indicated, similar to our results, limited transition from exclusive smoking to exclusive smokeless tobacco use [32].

In Sweden, both cigarette starters and snus starters were found, in contrast to our study, to have a low risk to end up as current smokers [31]. In USA, one study did not find any association between snus debut and later smoking [33] and another found little evidence of transition from one tobacco product in adolescence to another in adulthood [34].

Scandinavian studies among adults have supported a possible harm reduction effect of snus; A Swedish study found that men using both cigarettes and snus during their lifetime were likely to quit cigarettes and continue with snus only. The same research group found the availability of snus to contribute to the low Swedish rates of smoking among men [12, 13]. Lund et al. studied cigarette smoking in Norway in light of the availability of snus between 1985 and 2012, and found snus use to enhance the quit rates for smoking among adults [11]. The results were not replicated in USA, where transitions between cigarettes and smokeless tobacco was infrequent [35]. Also, smoking cessation for dual users was not different from that of exclusive smokers, and even when the dual users were more likely to have tried to quit, they were found to relapse more quickly than the smokers [36, 37].

In our study, the high rates of dual use with daily snus use and low frequent smoking in adulthood may be seen as a step on the way to exclusive snus use or non-use of tobacco. In line with this, the dual users in our cohort had a high probability to be snus only users in adulthood. However, widespread tobacco use (49%) among the young adults in our cohort is worrying. Declining cigarette smoking, but stable rates of overall tobacco use and poly-tobacco use among youth, are reported from USA [38]. A Norwegian study found a potential for harm reduction with snus, but also a tendency to combine non-daily smoking and snus use [14]. In our study, the adolescent dual users seem to be clearly more dependent with lower RR of becoming non-users of tobacco in adulthood than the corresponding smokers or snus users. Dual and poly-tobacco use has been associated with high risk adolescents and high levels of nicotine dependency in other countries as well [31, 38].

A main impression across studies is that adolescent tobacco users seem to be more likely than adults to progress from snus to smoking. The studies also show that transitions between tobacco products vary between countries and are probably influenced both by their relative availability, the pattern of use in peers, marketing strategies for sale, and by national tobacco policies [39, 40].

### Are snus users predisposed to smoking?

Snus users and smokers seem to have much of the same susceptibility to tobacco use, according to individual background factors. In this sense, one could speculate if adolescents who use snus might smoke if snus was not available [41].

Exclusive snus use in adolescence has been associated with psychosocial risk factors similar to smokers, but with healthier behaviour and higher academic orientation compared to smokers and dual users [1]. Similarly, the risk profile of snus users regarding social factors, lifestyle and health were found to lay between non-users of tobacco and smokers, being less favourable than those of non-users, but more advantageous than those of smokers [14, 18, 42]. Different risk profiles of snus users and smokers points to partly different user groups. In our study, this may explain a higher propensity of adolescent smokers than snus users to be smokers or dual users in young adulthood.

Nicotine dependence might explain a common propensity of future smoking as well as snus use. The quantities of delivered nicotine in snus are similar to cigarettes [7]. Easy access to sufficient amounts of nicotine from snus, as smoking in restaurants and bars was banned in 2004, may have influenced the transition to snus in our cohort period. In Norway, the snus prices being lower by about 75–80% of the cigarettes prices gives snus another preference [43]. The use of other tobacco products, as hookah and cigarillos, are almost non-existing in Norway [17]. Electronic cigarettes with nicotine have not yet entered the market for sale.

### Methodological considerations

The large full-scale population with a high participation rate as adolescents gave a representative sample in Young-HUNT1 at baseline. The broad range of demographic and behavioural measures in Young-HUNT1 allowed thorough examination of risk factors at baseline. Another strength was the long follow-up time and the possibility to examine the transitions into the more established tobacco use in young adulthood. The importance of including former smoking as baseline ovariates when studying predictors of future smoking has been addressed in several studies [27, 33]. By incorporating former tobacco use in the predictor variables at baseline, the importance of early tobacco use for later use and dependence is taken into account [39]. The validity of self-reported tobacco use has been demonstrated among adolescents and adults [44, 45]. However, we found some inconsistence in reported tobacco use between baseline and follow-up, which may indicate over reporting of occasional tobacco use among the adolescents at baseline. Young adults at follow-up may also have underreported earlier tobacco use in the direction of desirable behavior, or they may have forgotten about their tobacco use 11 years earlier. Those with the lowest levels of occasional use may have been more likely to forget about it. In an additional analysis where the inconsistent records were removed, we found an increase in the effect size for tobacco use transitions, indicating low levels of tobacco use among those with inconsistent answers.

One important limitation was the low participation rate in the age group 20–29 years in HUNT3. The low participation rate in HUNT3 among our cohort participants from Young-HUNT1 was possibly affected by the setting of large population studies and the inclusion of both eligible and not eligible (moved out of the county for education etc.) as basis for the follow-up in Young-HUNT. However, to improve the response rate, non-responders in HUNT3 received a short version of the questionnaire by mail including core questions on health and lifestyle [15]. Other strategies to recruit participants to HUNT3 included information to the entire population in different news channels. One main incentive of participating in HUNT3 may have been the benefit of a health check [16], obviously a bigger gain for older age groups than for the young men in our cohort. Participants lost to follow-up had higher prevalence of family members smoking and parental divorce than those in the study population, which may be indicators of lower socioeconomic status among non-participants [20]. A higher prevalence of smoking was found among those not attending follow-up, especially among the older age smokers. Corresponding differences were small for snus users and dual users. Thus, a selection of too few, and perhaps less vulnerable smokers in our study population may have taken place. One implication of this is possibly an underestimation of the transitions to smoking and dual use in adulthood, rather than the opposite.

Compared to national surveys, a relatively high prevalence of snus use was found at early stages in Nord-Trøndelag. In other aspects, we have no reason to believe that these data would differ much from national data.

For some purposes it is a limitation with cohort-data back to 1995–97 and 2006–08. Nevertheless, even when collected a decade back in time, our data have the benefits of following the adolescents into young adulthood in a time where smoking was still prevalent and snus use started to rise (Additional file 5). Norway was among the first countries to introduce new nicotine products with reduced harm potential [11]. Hence, this experience may be useful as a parallel to the recent introduction of e-cigarettes in many countries.

A small glance at the mainly smoking women in the same cohort showed nearly no increase in the prevalence of smoking from adolescence to adulthood, combined with a modest increase of snus use and dual use at low levels (Additional file 1). The men in our study population had similar smoking prevalence as the women in adulthood, dual use included.

## Conclusions

The adolescent snus users and dual users conferred a high risk of being tobacco users in young adulthood. The extensive use of snus among the young boys in our study is followed by persistent dual use and smoking into adulthood. The desired effect of snus in reducing smoking is not apparent, as tobacco use was escalating in men while fairly stable in women. This experience from a Norwegian population study reveals possible disadvantages of the access to new nicotine products.

## Supplementary information

**Additional file 1.** Key results for females. (DOCX 24 kb)

**Additional file 2.** Baseline characteristics for participants and non-participants to the cohort population. (DOCX 16 kb)

**Additional file 3.** Current tobacco use for participants and non-participants to the cohort population. (DOCX 16 kb)

**Additional file 4.** Baseline personality traits and school factors by tobacco use. Young men 13–19 years of age. (DOCX 15 kb)

**Additional file 5.** Daily smoking and snus use in Norway 1995–2010. Men and women 16–24 years. (DOCX 16 kb)

## Data Availability

Due to confidentiality, HUNT Research Centre limit storage of data outside HUNT databank, and has restrictions for researchers for handling of HUNT data files. However, precise information on all data exported to different projects are kept and there are no restrictions regarding data export given approval of applications to HUNT Research Centre, http://www.ntnu.edu/hunt/data.

## Abbreviations

CI: Confidence interval
Dual use: Current use of both snus and cigarettes
HUNT: The Nord-Trøndelag Health Study
RR: Relative risk
SD: Standard deviation
SE: Standard error
Snus: Smokeless tobacco
ST: Smokeless tobacco
Swedish snus: Smokeless tobacco
