# Supplementary material for: Impact of snus use in teenage boys on tobacco use in young adulthood; a cohort from the HUNT Study Norway
Source: BMC Public Health. 2019 Sep 13;19:1265. doi: 10.1186/s12889-019-7584-5 (PMC6743150; doi:10.1186/s12889-019-7584-5)
Supplement: Supplementary file 1 — Additional file 1. Key results for females. (DOCX 24 kb) [file 12889_2019_7584_MOESM1_ESM.docx]

Additional file 1. Key results for females

**Impact of smoking in teenage girls on tobacco use in young adulthood. A cohort from the HUNT Study Norway.**

**Background**

The male population in Norway used snus at an earlier stage than females (Additional file 5). This was the case for Norway as a whole, but also in our study from Trøndelag County. The men in our cohort had higher prevalence of snus use than of smoking, while the women in our cohort mostly were smokers. This may have been a disadvantage for the females, because of the lower harm potential of snus compared to cigarettes. After the millennium women slowly started to use snus in Norway. Thus, it is of importance to know how the tobacco use among the females changed during the 11 years between Young-HUNT1 in 1995-97 and HUNT3 in 2006-2008. The main objective was to find out how the tobacco use among the mainly smoking girls changed into adulthood. Did they quit smoking or not? To what degree did they start to use snus? Some key results are shown here for the female part of the cohort described in this study.

**Methods**

Because of low baseline prevalence of snus use among females, they were not included in the present study. 4463 girls (13-19 years) participated at baseline, 4354 participated with information about tobacco. Among those, 44% also participated at follow-up. The cohort population consisted of 1843 female participants with tobacco information at both time points. Missing values for both tobacco questions constituted 2% in adolescence and 4% in adulthood. The methods are described in the main study manuscript.

**Results**

Mean age for the 1843 participants in the cohort population was 16.2 (range 12.7-21.3) years in adolescence and 27.9 (range 22.8-33.0) years as young adults. While 22 % of the girls were current tobacco users in adolescence (Table 1), 29% were ever tobacco users (Table 2). Among the young women at follow-up, 31% were current tobacco users.

In contrast to the males, the female cohort participants were mainly smokers and the smoking prevalence more than doubled from adolescents aged 13-15 to those aged 16-19 (age groups not shown in Table 2). In addition, 2% were snus users and 2% were dual users of snus and cigarettes in the older age group at baseline. The female adolescent smokers had higher prevalence of parental divorce, family smoking and parental alcohol use than the non-smokers. Girls with vocational education plans and those not yet decided about education had higher smoking prevalence than those with plans for high school or university (Table 1).

The crude prevalence of ever tobacco use in adolescence and current tobacco use in young adulthood is shown in Table 2. Most of the adolescent smokers remained tobacco users as young adults, 43% of them were still smokers, and 10% of them were either snus users or dual users, while 47% used no tobacco as young adults. Hence, nearly every second baseline ever smoker had quit all tobacco as adults. The probability to quit was 2 in 5 for dual users and 2 in 3 for snus users. Relatively few had started to use snus, but 6% were current snus users and 3% were dual users at follow-up. The prevalence of current tobacco use in total was 31% among the young female adults, compared to 49% among the young male adults.

Adolescent smokers and dual users had a threefold likelihood to be smokers as adults (Table 3). The RRs of female adolescent ever smokers to be smokers (or dual users) in young adulthood, adjusted for age and family smoking, was 3.2 (CI 2.8-3.8). Very similar results were found for dual users with RR 3.3 (CI 2.3-4.6). Dual users also had a threefold likelihood, however with wide confidence intervals, to be snus only users in adulthood with RR 3.5 (1.6-7.5). The transition from adolescent smoking to snus only use in adulthood was relatively low (1.4, CI 0.9-2.2). The low risk of female snus users to be smokers in adulthood was non-significant (RR 0.3, CI 0.1-2.2), but baseline snus users had a fourfold risk, with wide confidence intervals, to still be snus users in adulthood (RR 4.1 CI 2.1-8.1). The high RR for adolescent female snus users to be tobacco free in adulthood was non-significant.

**Discussion**

Female adolescent smokers and dual users had a high risk of still being smokers or dual users in adulthood, and these transitions are similar to those found for the male participants. The relatively low and non-significant RR for a transition from smoking in adolescence to snus use in adulthood, was also similar to the results found for the male participants. Both baseline snus users and dual users conferred a high degree of uncertainty due to small numbers, but both groups had a high tendency to snus only use in young adulthood.

The strength in the transition from smoking to snus use is of interest in the debate of the harm reduction effect of snus use. In this cohort of women, we found similar results as for men, with little support for the transition from smoking in adolescents to snus use in adulthood. Another way to harm reduction is the transition from dual use to snus only use. Consistent with the results for men, we found that adolescent dual users had a high likelihood to be snus only users in adulthood. Hence, dual use may have been a way out of smoking for many smokers. On the other hand, male and female dual users have the same high likelihood to be smokers or dual users also in adulthood. Interestingly, the female dual users had a significantly higher likelihood to be non-users of tobacco in adulthood than the male dual users. Female smokers had a similar likelihood not to use any tobacco in adulthood as the male adolescent smokers and snus users.

**Conclusion**

The women were mainly smokers as adolescents and as adults. It was almost no increase in current smoking among women between baseline (20%) and follow-up (22%). The lack of increase may have been influenced by new restrictions, an increasing awareness of the harm of smoking and the general decreasing smoking trends after the millennium (Additional file 5).

Among females, total current tobacco use increased from 22% in adolescence to 31% in adulthood. The corresponding figures for male participants were an increase from 27% to 49%. This may indicate that the low prevalence of snus use and dual use among the girls at baseline mainly has been an advantage for the female participants.

Table 1. Sociodemographic and behavioural characteristics, by current tobacco use in adolescence*

|  | **Total** | **Snus use,**  **but not smoke** | **Smoking, but not snus** | **Dual use** | **No tobacco** | **p-value**** |
| --- | --- | --- | --- | --- | --- | --- |
| **Participants** | 1843 | 29 (2) | 360 (20) | 23 (1) | 1431 (78) |  |
|  |  |  |  |  |  |  |
| **Age** years, mean ± SD | 16.2 (± 1.8) | 16.6 (± 1.9) | 17.0 (± 1.6) | 17.4 (± 1.6) | 16.0 (±1.8) |  |
| 13-15 years, n (%) | 877 (100) | 11 (1) | 101 (12) | 4 (0) | 761 (87) |  |
| 16-19 years, n (%) | 966 (100) | 18 (2) | 259 (27) | 19 (2) | 670 (69) | <0.001 |
|  |  |  |  |  |  |  |
| **Parents living together** |  |  |  |  |  |  |
| Mother and father married/ living together, n (%) | 1500 (100) | 23 (2) | 264 (18) | 16 (1) | 1197 (80) |  |
| Mother and father divorced/ not living together, n (%) | 318 (100) | 6 (2) | 92 (29) | 7 (2) | 213 (67) | <0.001 |
|  |  |  |  |  |  |  |
| **Family smoking** |  |  |  |  |  |  |
| No family member smoke, n (%) | 904 (100) | 17 (2) | 120 (13) | 10 (1) | 757 (84) |  |
| Father *or* mother smokes, n (%) | 570 (100) | 9 (2) | 118 (21) | 6 (1) | 437 (77) |  |
| Father *and* mother smoke, n (%) | 272 (100) | 1 (0) | 81 (30) | 7 (3) | 183 (67) |  |
| Siblings *and/ or* others smoke, but no parent, n (%) | 91 (100) | 2 (2) | 38 (42) | 0 (0) | 51 (56) | <0.001 |
|  |  |  |  |  |  |  |
| **Parental alcohol use** |  |  |  |  |  |  |
| Have never seen parents drunk, n (%) | 647 (100) | 8 (1) | 76 (12) | 5 (1) | 558 (86) |  |
| Yes, a few times, n (%) | 708 (100) | 10 (1) | 159 (22) | 8 (1) | 531 (75) |  |
| Yes, sometimes a year, monthly or weekly, n (%) | 447 (100) | 11 (2) | 121 (27) | 10 (2) | 305 (68) | 0.001 |
|  |  |  |  |  |  |  |
| **Plans for own education** |  |  |  |  |  |  |
| Not yet decided, n (%) | 486 (100) | 3 (1) | 106 (22) | 3 (1) | 374 (77) |  |
| Vocational high school or similar, n (%) | 380 (100) | 5 (1) | 95 (25) | 5 (1) | 275 (72) |  |
| High school until 4 years, n (%) | 523 (100) | 10 (2) | 77 (15) | 6 (1) | 430 (82) |  |
| University, more than 4 years, n (%) | 402 (100) | 10 (2) | 73 (18) | 9 (2) | 310 (77) | <0.002 |

* All the tobacco use categories include both daily and occasional use. Variables with missing data include Parents living together (1%), Family smoking (0.3%), Parental alcohol use (2%), and Plans for own education (3%). ** p-value: test for independence between the socio-demographic and the tobacco variable at baseline.

Table 2. Tobacco use among females13-19 years in adolescence and 23-30 years in adulthood. Number (%). Study population, unadjusted.

|  |  | **CURRENT TOBACCO USE IN YOUNG ADULTHOOD** | | | | |
| --- | --- | --- | --- | --- | --- | --- |
| **EVER TOBACCO USE IN ADOLESCENCE** |  | No tobacco | Snus only | Smoke only | Dual use | All |
|  | No tobacco | 1016 (78.1) | 77 (5.9) | 179 (13.8) | 29 (2.2) | 1301 (100) |
|  | Snus only | 21 (72.4) | 7 (24.1) | 1 (3.4) | 0 | 29 (100) |
|  | Smoke only | 219 (47.2) | 21 (4.5) | 199 (42.9) | 25 (5.4) | 464 (100) |
|  | Dual use | 21 (42.9) | 6 (12.2) | 18 (36.7) | 4 (8.2) | 49 (100) |
|  | All | 1277 (69.3) | 111 (6.0) | 397 (21.5) | 58 (3.2) | 1843 (100) |

Table 3 females. Tobacco use in adulthood according to adolescent ever tobacco use 11 years earlier. RR (CI).

|  | Current smoking/dual use as young adults* | | Current snus only use as young adults* | | No tobacco use as young adults** | |
| --- | --- | --- | --- | --- | --- | --- |
|  | Adjusted for age | Adjusted for age and family smoking | \| Adjusted for age \| \| --- \| | Adjusted for age and family smoking | Adjusted for age | Adjusted for age and family smoking |
|  | N =1732 | N =1727 | N =1388 | N =1383 | N =1843 | N =1837 |
| Tobacco use at baseline: |  |  |  |  |  |  |
| No tobacco | ref. | ref. | ref. | ref. | ref. | ref. |
| Smoking | 3.48 (2.98-4.05) | 3.24 (2.77-3.79) | 1.41 (0.88-2.24) | 1.39 (0.87-2.24) | 0.57 (0.52-0.63) | 0.59 (0.53-0.65) |
| Dual use | 3.56 (2.55-4.97) | 3.26 (2.34-4.55) | 3.55 (1.67-7.55) | 3.50 (1.63-7.49) | 0.52 (0.37-0.72) | 0.53 (0.38-0.74) |
| Snus use | 0.33 (0.05-2.22) | 0.34 (0.05-2.22) | 4.06 (2.07-7.94) | 4.14 (2.11-8.13) | 0.87 (0.70-1.08) | 0.85 (0.69-1.07) |

* Versus no current tobacco use. ** Versus any tobacco use
