# Supplementary material for: Impact of snus use in teenage boys on tobacco use in young adulthood; a cohort from the HUNT Study Norway
Source: BMC Public Health. 2019 Sep 13;19:1265. doi: 10.1186/s12889-019-7584-5 (PMC6743150; doi:10.1186/s12889-019-7584-5)
Supplement: Supplementary file 2 — Additional file 2. Baseline characteristics for participants and non-participants to the cohort population. (DOCX 16 kb) [file 12889_2019_7584_MOESM2_ESM.docx]

Additional file 2. Baseline characteristics for participants and non-participants to the cohort population. Percent
