# Supplementary material for: Impact of snus use in teenage boys on tobacco use in young adulthood; a cohort from the HUNT Study Norway
Source: BMC Public Health. 2019 Sep 13;19:1265. doi: 10.1186/s12889-019-7584-5 (PMC6743150; doi:10.1186/s12889-019-7584-5)
Supplement: Supplementary file 4 — Additional file 4. Baseline personality traits and school factors by tobacco use. Young men 13–19 years of age. (DOCX 15 kb) [file 12889_2019_7584_MOESM4_ESM.docx]

Additional file 4. Baseline personality traits and school factors by tobacco use. Young men 13-19 years of age.*

|  | Total, mean (CI) | Snus, but no smoke | Smoke, but no snus | Dual use | No tobacco |  |
| --- | --- | --- | --- | --- | --- | --- |
| Neurotic personality traits, mean score (CI), n=1269 | 2.43 (2.34-2.53) | 2.66 (2.36-2.95) | 2.76 (2.41-3.11) | 3.07 (2.77-3.36) | 2.29 (2.18-2.40) |  |
| Extrovert personality traits, mean score (CI), n=1220 | 4.50 (4.42-4.57) | 4.84 (4.64-5.04) | 4.82 (4.58-5.06) | 5.02 (4.79-5.25) | 4.35 (4.25-4.44) |  |
| Psychotic personality traits, mean score (CI), n=1249 | 0.85 (0.80-0.91) | 0.84 (0.68-1.00) | 0.92 (0.73-1.11) | 1.24 (1.04-1.44) | 0.81 (0.74-0.87) |  |
| School factor; gratuitous, mean score (CI), n=1305 | 7.65 (7.60-7.71) | 7.57 (7.38-7.76) | 7.76 (7.55-7.98) | 8.05 (7.83-8.28) | 7.61 (7.55-7.67) |  |
| School factor; restless, quarrelsome,  mean score (CI), n= 1305 | 4.68 (4.61-4.76) | 4.91 (4.66-5.16) | 5.08 (4.80-5.37) | 5.54 (5.25-5.84) | 4.51 (4.43-4.59) |  |
| School factor; well-adjusted, positive,  mean score (CI), n=1279 | 6.55 (6.48-6.61) | 6.49 (6.30-6.68) | 6.12 (5.84-6.40) | 6.32 (6.09-6.55) | 6.63 (6.55-6.70) |  |

* The total number of participants in the cohort is 1346. Because of missing values, participants varied between 1220 and 1305 for the shown variables.
