# Supplementary material for: Impact of snus use in teenage boys on tobacco use in young adulthood; a cohort from the HUNT Study Norway
Source: BMC Public Health. 2019 Sep 13;19:1265. doi: 10.1186/s12889-019-7584-5 (PMC6743150; doi:10.1186/s12889-019-7584-5)
Supplement: Supplementary file 5 — Additional file 5. Daily smoking and snus use in Norway 1995–2010. Men and women 16–24 years. (DOCX 16 kb) [file 12889_2019_7584_MOESM5_ESM.docx]

Additional file 5 Daily smoking and snus use in Norway 1995-2010. Men and women 16-24 years. Percent

Source: Statistics Norway and Norwegian Institute of Public Health
